# Alternative Cell Death Pathways and Cell Metabolism

**DOI:** 10.1155/2013/463637

**Published:** 2013-01-21

**Authors:** Simone Fulda

**Affiliations:** Institute for Experimental Cancer Research in Pediatrics, Goethe-University Frankfurt, Komturstraße 3a, 60528 Frankfurt, Germany

## Abstract

While necroptosis has for long been viewed as an accidental mode of cell death triggered by physical or chemical damage, it has become clear over the last years that necroptosis can also represent a programmed form of cell death in mammalian cells. Key discoveries in the field of cell death research, including the identification of critical components of the necroptotic machinery, led to a revised concept of cell death signaling programs. Several regulatory check and balances are in place in order to ensure that necroptosis is tightly controlled according to environmental cues and cellular needs. This network of regulatory mechanisms includes metabolic pathways, especially those linked to mitochondrial signaling events. A better understanding of these signal transduction mechanisms will likely contribute to open new avenues to exploit our knowledge on the regulation of necroptosis signaling for therapeutic application in the treatment of human diseases.

## 1. Introduction

There are several forms of cell death in mammalian cells, among them apoptosis and necrosis as the two best characterized and most intensively studied modes of cell death [1]. Apoptosis is characterized by a series of programmed events, including membrane blebbing, caspase activation, and internucleosomal DNA fragmentation [1]. In contrast to apoptosis, necrosis represents a form of cell death that typically lacks the activation of caspases, while it involves swelling of mitochondria, irreversible damage to cellular membranes, eventually leading to spilling of the intracellular content into the surrounding environment [1]. In addition, a regulated form of necrosis, that is, necroptosis, has recently been identified that proceeds in a programmed and controlled manner [2]. Necroptosis refers to RIP1- and/or RIP3-dependent regulated necrosis [1]. A better understanding of the molecular mechanisms that regulate necroptosis signal transduction may open new perspectives for targeted modulation and therapeutic exploitation of necroptosis signaling. This paper will focus on the crosstalk between necroptosis and metabolic signaling events, in particular redox signaling.

## 2. Necroptosis Signaling

There are various stimuli that can engage necroptosis, including ligands of the death receptor family such as TNF*α*, chemical or physical damage, or hypoxic conditions [2]. Since necroptosis represents a programmed form of cell death, there are clearly delineated signal transduction cascades that eventually lead to the demise of the cell. 

For example, in TNF*α*-triggered necroptosis, binding of the death receptor ligand TNF*α* to cognate plasma membrane receptors on the cell surface, that is, TNF receptor 1 (TNFR1) as the main receptor for TNF*α*, engages an intracellular signaling machinery [3]. This involves the recruitment of adaptor molecules such as TRADD signaling molecules such as RIP1, cIAP1, cIAP2, TRAF2, and TRAF5 to the cytoplasmic part of TNFR1. The recruitment of the signaling molecules leads to the formation of the so-called complex I, a multimeric protein complex that leads to the polyubiquitination of RIP1 by cIAP proteins. Following its polyubiquitination, RIP1 can serve as docking site for TAK1, TAB2 and TAB3 which results in the activation of the transcription factor NF-*κ*B. Upon binding of TNF*α*, TNFR1 is internalized in order to terminate signal transduction via the ligated membrane-bound receptor which subsequently leads to a change in the molecular composition of the interacting signaling proteins. This enables the formation of the so-called TNFR1 complex II which contains core components such as FADD, RIP1, and caspase-8 and serves as a platform for caspase-8 activation and initiation of apoptosis. However, if caspase-8 is insufficiently activated in this complex, an alternative complex can be formed, that is, the so-called necrosome complex [2]. This necrosome complex contains FADD, RIP, and in addition also RIP3 and is critically required for the initiation of necroptotic cell death. Since RIP1 can be a part of cell death and survival signaling complexes, posttranslational modifications of RIP1 ensure that fine tuning of RIP1 functions in apoptosis versus necroptosis versus NF-*κ*B signaling is achieved. Accordingly, the ubiquitination of RIP1 favors the formation of the cell death-initiating platforms, whereas ubiquitinated RIP is a component of the NF-*κ*B-signaling cascade [2]. Upon the formation of the necrosome complex, RIP1 and RIP3 become phosphorylated and activated which in turn engages downstream signaling events that eventually lead to necroptotic cell death [4]. Of note, these signaling networks also involve several crosstalks with cellular metabolic pathways.

## 3. Regulation of Necroptosis by Redox Signaling

The regulatory mechanisms that are involved in the control of the initiation and propagation of necroptosis are still largely unknown. There is accumulating evidence that redox processes play an important role in the regulation of necroptosis. For example, the production of ROS is rapidly increased during the early stages of necroptosis [5, 6] indicating that ROS generation may be involved in mediating necroptosis. Indeed, it has been shown that the inhibition of the production of reactive oxygen species (ROS) also reduces the induction of necroptosis [5, 7]. 

What are the generator systems for ROS production during necroptosis? There are several potential intracellular sites that can contribute to ROS production in the course of necroptosis. In principle, they can be divided into mitochondrial generator systems and extramitochondrial sites for ROS production. Within mitochondria, complexes I and III of the respiratory chain are considered as main sites for ROS production during programmed cell death [8]. In addition, the mitochondrial adenine-nucleotide translocates (ANT) can contribute to ROS production in the course of cell death. ANT is localized at the inner mitochondrial membrane and responsible for the exchange of ADP against ATP [9]. The inhibition of ANT leads to a decrease of ADP levels with concomitant increase in ATP in the mitochondrial matrix, which in turn reduces the activity of the ATP synthase and hyperpolarization of the mitochondrial membrane potential, thereby favoring the production of ROS [10]. Since RIP1 has been described to negatively regulate ANT activity, it is tempting to speculate that elevated RIP1 activity during necroptosis may inactivate ANT, thereby favoring the production of ROS species. 

As far as extramitochondrial sources of ROS are concerned, NOX NADPH oxidases represent the main sources of ROS production in the extramitochondrial compartment [11]. It is interesting to note that the prototypic necroptosis stimulus TNF*α* has recently been reported to increase the activity of NOX1 complex via a mechanism involving RIP1 [12]. TNFR1 complex I has also been reported to serve as platform, which enables the docking of the NADPH oxidase NOX1 at the plasma membrane, thereby promoting the generation of ROS [12]. This involves the TNF*α*-stimulated association of NADPH oxidase organizer 1 (NOXO1) subunit with RIP1, TRADD and riboflavin kinase, which in turn results in ROS production via NOX1 [12].

## 4. ROS Species as Executioners of Necroptosis

Already twenty years ago the execution of necrotic cell death has been associated with ROS species as critical mediators of cell death. It was reported that complex I-mediated production of ROS species is required for necrotic cell death in response to TNF*α* stimulation [8]. In addition, ROS production within the mitochondria has been associated with structural changes and damage to organelles such as mitochondria and the endoplasmic reticulum [8, 13]. In addition to mitochondrial ROS, the generation of ROS from extramitochondrial sources, for example, via the plasma membrane-associated NADPH oxidase NOX1, has been shown to mediate necrotic cell death upon stimulation with TNF*α* [12]. ROS generation by NOX1 might not only result in lipid peroxidation and membrane damage but may also engage a feed-forward amplification loop to trigger further ROS production via the mitochondrial respiratory chain. Another amplification loop might involve the lysosomal compartment where hydrogen peroxide can interact with ferrous ions to produce hydroxyl radical (Fenton reaction), a highly reactive ROS species [14]. Such amplification loops can lead to the overproduction of ROS, for example, at the mitochondrial respiratory chain. This bears the danger of a lethal vicious cycle eventually resulting in the generation of reactive nitrogen species (RNS). RNS species can function as oxidants to produce protein or lipid oxidation, thereby altering protein function and causing membrane damage [15].

Moreover, ROS generation has also been linked to mitochondrial bioenergetics. To this end, advanced glycation end products (AGE) which are generated as the result of several chemical reactions in response to elevated levels of extracellular glucose have been reported to bind to receptors on the cell surface and to promote ROS production [16].

## 5. Bioenergetic Regulation of Necroptosis

Apoptosis and necrosis not only represent morphologically two distinct forms of cell death but also from the aspect of bioenergetics demands. The initial characterization of apoptotic and necrotic cell death has revealed that intracellular adenosine triphosphate (ATP) content represents a central regulator in the decision on the mode of cell death, that is, apoptosis and necrosis. Accordingly, human T-cells have been reported to switch from apoptosis in response to CD95 stimulation or treatment with staurosporine towards necrosis upon depletion of ATP [17]. In this model, the generation of ATP by either the mitochondrial respiratory chain or by glycolysis was shown to be necessary to provide the energetic supply to execute apoptosis via DNA fragmentation [17]. The addition of extramitochondrial ATP, for example, by repletion of glucose, resulted in restored ability of T cells to undergo apoptotic cell death [17]. Subsequently, different steps in the apoptotic signaling cascade were shown to depend on sufficient supply of bioenergetics substrates and ATP consumption, for example, activity of the translational machinery, protein degradation via the ubiquitin proteasome system, and activity of DNA repair enzymes such as PARP1 [18–20]. PARP1 has been described to play an important role in the metabolic regulation of cell death. PARP1 is localized in the nucleus and can sense DNA damage, which leads to the overactivation of PARP1 when DNA damage is extensive [21, 22]. PARP overactivation then causes depletion of NAD and ATP and an acute bioenergetic depletion, which promotes the release of apoptosis-inducing factor (AIF) from the interspace of mitochondria and its translocation to the nuclear compartment [23]. Within the nucleus, AIF is supposed to be required for large-scale DNA fragmentation in a caspase-independent manner [23]. This type of PARP1-dependent necrotic cell death has been implicated in several pathophysiological conditions, for example, upon cerebral ischemia, oxygen-glucose depletion, or exposure to alkylating DNA-damaging agents [21, 22, 24].

The mitochondrial permeability transition pore complex (PTPC) represents another multimeric protein complex that has been implied in the bioenergetic control of several cell death modalities [25]. The PTPC is composed of ANT, an integral protein of the inner mitochondrial membrane that is responsible for the exchange of ATP with ADP across mitochondrial membranes [9], voltage-dependent anion channel (VDAC), a protein of the outer mitochondrial membrane [26], and cyclophilin D, which is localized to the mitochondrial matrix [27]. The activity of VDAC is tightly controlled by various mechanisms including proteins of the Bcl-2 family, ions, and metabolites. The key role of VDAC in the regulation of necrotic cell death is underlined by genetic evidence showing that mice deficient in cyclophilin D are protected against ischemic injury [22, 28].

Downstream of the RIP1/RIP3 necrosome complex, metabolic pathways including glycogenolysis, and glutaminolysis have also been implicated to mediate signaling events during necroptosis. RIP3 has been shown to promote the activity of several metabolic enzymes including glycogen phosphorylase (PYGL), glutamate-ammonia ligase (GLUL), and glutamate dehydrogenase 1 (GLUD1) [6]. PYGL is a key enzyme in the catabolic metabolism of glycogen causing the conversion of glycogen into glucose-1-phosphate which in turn can be modified into glucose-6-phosphate, a substrate for glycolysis that can promote the generation of ROS via mitochondrial metabolic burst. In addition, RIP3 was shown to activate GLUL and GLUD1 [6], a cytosolic and mitochondrial enzyme, respectively, involved in glutaminolysis. Enhanced glutaminolysis can engage the Krebs cycle, eventually contributing to increased ROS generation. Thus, RIP3-dependent changes in glycogenolysis and glutaminolysis can result in enhanced energy metabolism and increased production of ROS.

## 6. Conclusions

Since its discovery, necroptosis has evolved as one of the key programmed cell death pathways in mammalian cells, which is deregulated in numerous human diseases. Thanks to enormous research efforts, considerable advances have been achieved in the identification of the signal transduction steps and control mechanisms that are involved in the control of necroptosis signaling pathways. Metabolic events including redox signaling represent critical events that are involved in the regulation of necroptosis signaling. Further insights into the fine-tuning of signaling processes will likely pave the avenue towards novel opportunities to target specific intervention points within in the necroptosis signaling cascade in order to achieve therapeutic benefits.
